# LXR agonist inhibits inflammation through regulating MyD88 mRNA alternative splicing

**DOI:** 10.3389/fphar.2022.973612

**Published:** 2022-10-14

**Authors:** Ni Li, Yan Li, Xiaowan Han, Jing Zhang, Jiangxue Han, Xinhai Jiang, Weizhi Wang, Yang Xu, Yanni Xu, Yu Fu, Shuyi Si

**Affiliations:** ^1^ State Key Laboratory of Bioactive Substances and Function of Natural Medicines, Institute of Materia Medica, Chinese Academy of Medical Sciences, Beijing, China; ^2^ NHC Key Laboratory of Biotechnology of Antibiotics, Institute of Medicinal Biotechnology, Chinese Academy of Medical Sciences and Peking Union Medical College, Beijing, China; ^3^ College of Food Science and Biology, Hebei University of Science and Technology, Shijiazhuang, Hebei

**Keywords:** LXR, inflammation, MyD88, alternative splicing, multi-target, TLR4

## Abstract

Liver X receptors (LXRs) are important regulators of cholesterol metabolism and inflammatory responses. LXR agonists exhibit potently anti-inflammatory effects in macrophages, which make them beneficial to anti-atherogenic therapy. In addition to transrepressive regulation by SUMOylation, LXRs can inhibit inflammation by various mechanisms through affecting multiple targets. In this study, we found that the classic LXR agonist T0901317 mediated numerous genes containing alternative splice sites, including myeloid differentiation factor 88 (MyD88), that contribute to inflammatory inhibition in RAW264.7 macrophages. Furthermore, T0901317 increased level of alternative splice short form of MyD88 mRNA by down-regulating expression of splicing factor SF3A1, leading to nuclear factor κB-mediated inhibition of inflammation. In conclusion, our results suggest for the first time that the LXR agonist T0901317 inhibits lipopolysaccharide-induced inflammation through regulating MyD88 mRNA alternative splicing involved in TLR4 signaling pathway.

## 1 Introduction

Atherosclerosis, the underlying pathological process in cardiovascular disease, is caused by multiple factors (Bruikman et al., 2017). In addition to metabolic disorders, it is now well recognized that macrophage inflammation is an important trigger of atherosclerosis (Libby, 2007). During atherogenesis, macrophages in the artery wall become overloaded with modified lipoproteins, such as oxidized low-density lipoprotein, and produce high levels of inflammatory cytokines, leading to leukocyte recruitment and plaque development (Wong et al., 2012).

Toll-like receptor (TLR) pathways are involved in initiation of inflammatory plaques and progression of atherogenesis (Afonina et al., 2017). After being sensed by lipopolysaccharide (LPS)-binding protein, LPS binds to TLR4 receptors on macrophage membranes. Subsequently, several crucial adaptor proteins are recruited, such as myeloid differentiation factor 88 (MyD88), followed by phosphorylating interleukin one receptor-associated kinase 4 (IRAK4) to activate downstream nuclear factor-κB (NF-κB) signaling. Furthermore, activation of these proteins leads to the production of many pro-inflammatory cytokines, such as tumor necrosis factor α (TNFα), interleukin 1β (IL-1β), and IL-6 (Miller et al., 2005; Libby, 2007; Wang et al., 2020). Beyond regulation of the NF-κB pathway at a transcriptional level, alternative splicing of mRNAs encoding several proteins related to NF-κB signaling (including MyD88, IRAK2, and IκB kinase ε) contributes to the inflammatory response (Burns et al., 2003; Koop et al., 2011). Therefore, there are multiple genes in TLR4 signaling that can serve as potential targets for anti-inflammation.

MyD88, an adaptor of TLR4 signaling, exists in two alternative splice forms (MyD88-L and MyD88-S) that perform opposing functions (Burns et al., 2003; Mendoza-Barbera et al., 2009). Moreover, MyD88-S splicing isoform is reported to exert a dominant-negative effect, leading to the termination of downstream NF-κB signaling (Burns et al., 2003). Lesly et al. (De Arras and Alper, 2013) found that MyD88-S mRNA levels could be regulated by splicing factor 3A subunit 1 (SF3A1), a mRNA splicing factor that functions in TLR-mediated signaling pathway to control MyD88-S production. In particular, repression of SF3A1 diminished the LPS-induced production of inflammatory cytokines IL-6 and TNFα (De Arras et al., 2013). Therefore, SF3A1 undoubtedly serves as an important regulator of TLR-mediated inflammatory processes. However, how specific regulation of SF3A1 takes place in LPS-induced inflammation is unclear.

Nuclear receptors liver X receptors (LXRs) are ligand-activated transcription factors expressed in numerous immune cell types, including macrophages (Kiss et al., 2013; Rasheed and Cummins, 2018). LXR transcriptional activity can be induced by certain natural oxysterols and synthetic agonists, such as T0901317 and GW3965 (Schultz et al., 2000; Collins et al., 2002). Therefore, LXR play an important role in different diseases because it has lots of target genes as a crucial transcription factor. Furthermore, pathological and pharmacological studies demonstrate that LXRs function as a notable signaling crosslink between inflammation and lipid metabolism (Schulman, 2017). LXR agonists have been shown to exhibit potently anti-atherogenic effects, which are attributed to their ability to inhibit inflammation and regulate cholesterol metabolism through regulating various target genes (Calkin and Tontonoz, 2010).

Previous studies indicate that LXR agonists can significantly limit the transcription of pro-inflammatory genes [including IL-6, IL-1β, C-C motif chemokine 2 (MCP-1), cyclooxygenase-2 (COX-2), TNFα, and inducible nitric oxide synthase (iNOS)] mediated by NF-κB or activating protein 1 without directly binding to DNA, in a process named transrepression (Ghisletti et al., 2009). SUMOylation of LXR upon ligand binding and clearance of nuclear corepressor are the molecular mechanisms underlying the inhibitory effect of LXR on LPS-induced inflammatory activation (Ogawa et al., 2005; Geiger et al., 2020). Additionally, multiple mechanisms of LXR agonists in inflammatory responses have been reported in the past (Jang et al., 2016; Wang et al., 2017). For example, activation of LXR inhibits signaling from TLRs to their downstream NF-κB and MAPK effectors through regulating LXR target gene-ABCA1 to influence membrane lipid organization (Ito et al., 2015). Although the effects of LXR agonists on cell membrane TLR4 and downstream NF-κB signaling are well established, the mechanism by which LXR agonists affect intermediate proteins involved in LPS-induced inflammation, such as MyD88, remains unknown.

In this study, T0901317 was found to mediate numerous genes containing alternative splice sited, such as MyD88. Moreover, T0901317 increased the mRNA levels of MyD88-S splicing isoform by down-regulating SF3A1 expression, leading to NF-κB-mediated inhibition of inflammation in RAW264.7 macrophages. Overall, we found that the LXR agonist T0901317 exhibited potently anti-inflammatory effect by regulating LPS-induced TLR4 signaling, and determined that the mechanism was at least partly attributed to regulation of MyD88 mRNA alternative splicing regulation through SF3A1.

## 2 Materials and methods

### 2.1 Reagents

T0901317, LPS and PMSF were purchased from Sigma-Aldrich (St. Louis, MO, United States). Dulbecco’s Modified Eagle’s Medium (DMEM) was purchased from Hyclone (Cytiva, MA, United States). BCA protein assay kit and fetal bovine serum (FBS) were purchased from Thermo Fisher Scientific (Carlsbad, CA, United States). RIPA lysis buffer, protease inhibitor and phosphatase inhibitor cocktail were purchased from Applygen Technologies (Beijing, China).

### 2.2 Cell culture and treatment

RAW264.7 macrophages were obtained from ATCC (Manassas, United States). The cells were cultured in DMEM with 10% FBS in a humidified incubator containing CO_2_ (5%) at 37°C. RAW264.7 macrophages were seeded at 4×10^5^ cells/mL on 6-well plates. When the cells reached 90% confluence, the medium was changed and the cells were treated with T0901317 at various concentrations.

For RNA extraction, cells were incubated in serum-free DMEM with T0901317 for 18 h, followed by stimulating with 200 ng/mL LPS for 3 h additionally. While in enzyme linked immunosorbent assay (ELISA) and western blot analysis, cells were incubated in serum-free DMEM with 200 ng/mL LPS and T0901317 for 18 h, then the culture medium and cells were collected for ELISA and western blot separately.

### 2.3 Real-time quantitative PCR and semi-quantitative RT-PCR

Total RNA was extracted from the cells using TransZol Up Plus RNA Kit (TransGen Biotech, Beijing, China) according to the manufacturer’s instructions, followed by cDNA synthesis with the reverse transcriptional kit (TransGen Biotech), then the products were used for real-time quantitive PCR (qPCR) and semi-quantitative RT-PCR (RT-PCR) experiments respectively. qPCR with SYBR (CoWin Biosciences, China) detection was performed on the FTC-3000 Real-Time PCR Detection System (Funglyn Biotech Inc., Canada). RT-PCR analysis was performed with mixture of the cDNA product, primers, 2×polymerase mix (TransGen Biotech) for 40 cycles, then the PCR products were separated with 2% agarose gel at 90 V for 1.5 h in TAE buffer. The images were obtained from the GelDoc XR system (Biorad, Hercules, CA, United States) and analyzed using ImageJ software. The mRNA levels of all genes were normalized for glyceraldehyde-phosphate dehydrogenase (GAPDH) levels, and the quantitative measurements were calculated by the ΔΔCt method. The sequences of all the primers are listed in “Table 1”.

**TABLE 1:** Primers for real-time quantitative PCR and semi-quantitative RT-PCR.

Gene	Forward primers	Reverse primers
mouse GAPDH	5′- CAA​TGT​GTC​CGT​CGT​GGA​TCT -3′	5′- GTC​CTC​AGT​GTA​GCC​CAA​GAT​G -3′
mouse IL-1β	5′- AGA​AGC​TGT​GGC​AGC​TAC​CTG -3′	5′- GGA​AAA​GAA​GGT​GCT​CAT​GTC​C -3′
mouse IL-6	5′- CTG​CAA​GAG​ACT​TCC​ATC​CAG​TT -3′	5′- GAA​GTA​GGG​AAG​GCC​GTG​G -3′
mouse MYD88-L (qPCR)	5′- CCA​CCC​TTG​ATG​ACC​CCC​TAG​GAC​AAA​C -3′	5′- GTC​TGT​TCT​AGT​TGC​CGG​ATC​ATC​TCC​TGC​AC -3′
mouse MYD88-S (qPCR)	5′- GGA​GCT​GAA​GTC​GCG​CAT​CGG​ACA​AAC -3′	5′- GTC​TGT​TCT​AGT​TGC​CGG​ATC​ATC​TCC​TGC​AC -3′
mouse MYD88 (RT-PCR)	5′- TTG​TTG​GAT​GCC​TGG​CAG​GGG​CGC​TCT​GGC-3′	5′- CAC​GGT​CGG​ACA​CAC​ACA​ACT​TAA​GCC​GAT​AGT​C-3′
mouse SF3A1	5′-GGA​CCA​GGT​TTG​TTA​CCG​AGT-3′	5′-CCA​GTT​GGT​AAC​AAT​GCC​ATG​T-3′
mouse LXRα	5′-ACA​GAG​CTT​CGT​CCA​CAA​AAG-3′	5′-GCG​TGC​TCC​CTT​GAT​GAC​A-3′
mouse LXRβ	5′-ATG​TCT​TCC​CCC​ACA​AGT​TCT-3′	5′-GAC​CAC​GAT​GTA​GGC​AGA​GC-3′
mouse TLR4	5′-ATG​GCA​TGG​CTT​ACA​CCA​CC-3′	5′-GAG​GCC​AAT​TTT​GTC​TCC​ACA-3′

### 2.4 Western blot analysis

The cells were harvested and lysis in RIPA buffer supplemented with 1 mM PMSF, protease inhibitor and phosphatase inhibitor cocktail. The cell lysates were quantified using BCA protein assay kit and the Western Blot assays were carried out as previously described (Li et al., 2014). The following primary antibodies were used: mouse anti-GAPDH (1:4000, TransGen Biotech), mouse anti-β-Tubulin (1:4000, Proteintech Group, IL, United States), rabbit anti-COX-2 (1:1000, Proteintech), rabbit anti-iNOS (1:1000, Abcam, Cambridge, United Kingdom), mouse anti-TLR4 (1:2000, Proteintech), rabbit anti-SF3A1 (1:2000, Proteintech), rabbit anti-NF-κB p65 (1:500, Cell Signaling Technology), rabbit anti-phospho-NF-κB p65 (1:1000, Cell Signaling Technology), rabbit anti-IκBα (1:1000, Proteintech), rabbit anti-phospho-IκBα (1:1000, Bioworld Technology, Bloomington, United States). The protein bands were quantified by ImageJ Software, and normalized to GAPDH protein levels.

### 2.5 ELISA

After treating with T0901317 and LPS, the cell culture medium was collected. Then IL-1β and IL-6 cytokine levels were determined using ELISA commercial kits according to manufacturer’s instructions (ExCell Bio, Shanghai, China).

### 2.6 siRNA transfection

siRNA against mouse LXRα (forward: 5ʹ-GUG​UCA​UCA​AGG​GAG​CAC​GC UAUGUTT-3ʹ, reverse 5 ʹ- ACA​UAG​CGU​GCU​CCC​UUG​AUG​ACA​CTT-3′), LXRβ (forward: 5ʹ-GUCCACCAUUGAGAU CAUGUUGCUATT-3ʹ, reverse 5 ʹ- UAGCAA CAU​GAU​CUC​AAU​GGU​GGA​CTT-3′), mouse SF3A1 (forward: 5ʹ-GAAGAGACA GCC​AUU​GGU​AAG​AAG​ATT-3ʹ, reverse 5 ʹ- UCUUCUU ACCAAUGGCUGUCU CUUCTT-3′) and negative control siRNA (A06001) were used. All the siRNA were purchased from GenePharma (Shanghai, China). RAW264.7 macrophages were seeded at a density of 2 × 10^5^ cells/mL on 12-well plates. Briefly, 100 nM of siRNA (final concentration) was transfected into cells using Lipofectamine RNAi MAX transfection reagent (5 µL/well; Invitrogen, Carlsbad, CA, United States) according to the manufacturer’s instructions. After 24 h of transfection, cells were treated with serum-free DMEM for 18 h in the absence or presence of T0901317. Finally, cells were treated according to Section 2.2, and mRNA and protein levels were examined by qPCR and western blot assays, respectively.

### 2.7 RNA sequencing

RAW264.7 macrophages were pre-treated with T0901317 (1 μM) for 18 h, followed by stimulating with or without LPS (1 μg/mL) for 3 h, then total RNA was prepared as described in “Materials and methods” section. The libraries were constructed using the TruSeq Stranded mRNA LT Sample Prep Kit (Illumina, RS-122-2101), following the manufacturer’s instructions. RNA sequencing was performed on the Illumina platform (HiSeqTM 2500, Illumina, Shanghai OE Biotech. Co., Ltd.). The DEGs were identified based on the following criteria: *p* < 0.05 and fold changes <0.5 or >2. The alternatively splicing analysis of differentially regulated transcripts isoforms or exons was performed using ASprofile.

### 2.8 SF3A1 gene transfection

Overexpressed DNA transfection was used to upregulate SF3A1 expression levels. The pCMV3-Flag-SF3A1 expression plasmid (containing mouse SF3A1 gene ORF cDNA) was purchased from Sino Biological Company (Beijing, China). RAW264.7 macrophages were seeded at a density of 5 × 10^5^ cells/mL on 6-well plates. When reached to 80% confluent, the cells were transfected with or without pCMV3-Flag-SF3A1 plasmid DNA (3,000 ng/well) using Lipofectamine 2000 (Invitrogen) according to the manufacturer’s instructions. After 24 h of transfection, cells were treated with serum-free DMEM for 18 h in the absence or presence of T0901317. Finally, cells were treated according to Section 2.2, and mRNA and protein levels were examined by qPCR and western blot assays, respectively.

### 2.9 Statistical analysis

Results in this paper are shown as means ± SEM. Statistical significance was determined by one-way ANOVA or *t*’-test (GraphPad Prism 5.01 software). Value of *p* < 0.05 was considered to be statistically significant.

## 3 Results

### 3.1 T0901317 reduces LPS-induced inflammatory cytokines levels *via* LXR activation in RAW264.7 macrophages

**FIGURE 1:**
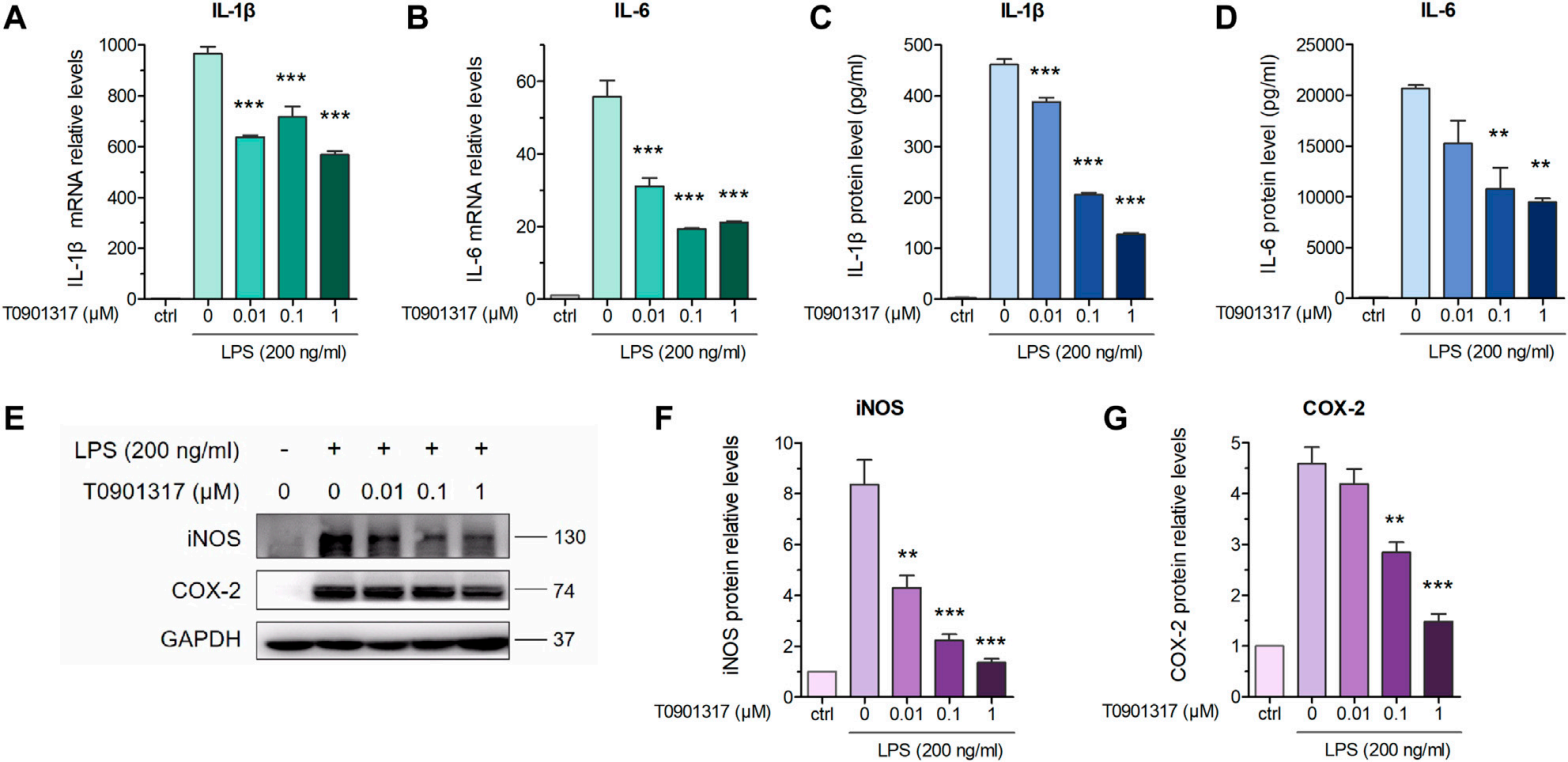
T0901317 reduces LPS-induced inflammatory cytokines levels in RAW264.7 macrophages. **(A,B)** RAW264.7 macrophages were pretreated with T0901317 (0, 0.01, 0.1, or 1 μM) for 18 h, then stimulated with or without LPS (200 ng/mL) for 3 h. IL-1β and IL-6 mRNA levels were measured by qPCR. **(C,D)** Following exposure of RAW264.7 macrophages to LPS (200 ng/mL) and T0901317 (0, 0.01, 0.1, or 1 μM) for 18 h, IL-1β and IL-6 protein levels were measured by ELISA kits. **(E–G)** Cells were exposed to LPS (200 ng/mL) and T0901317 (0, 0.01, 0.1, or 1 μM) for 18 h, then protein levels of iNOS, COX-2 were measured by western blot. All mRNA and protein levels were normalized to GAPDH. Similar results were obtained in three independent experiments. Values are expressed as means ± SEM and were analyzed by one-way ANOVA. ^**^ *p* < 0.01, ^***^ *p* < 0.001.

**FIGURE 2:**
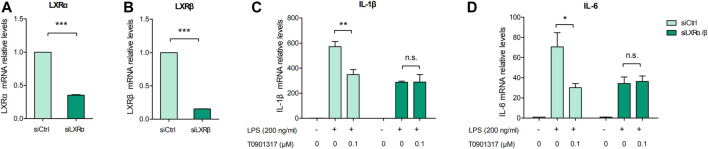
T0901317 reduces inflammatory cytokines levels *via* LXR activation. **(A–D)** RAW264.7 macrophages were transfected with negative control siRNA or LXRα/β siRNA for 24 h, followed by pretreating with T0901317 (0 or 0.1 μM) for 18 h, then stimulated with or without LPS (200 ng/mL) for 3 h. LXRα, LXRβ, IL-1β and IL-6 mRNA levels were measured by qPCR. All mRNA levels were normalized to GAPDH. Similar results were obtained in three independent experiments. Values are expressed as means ± SEM and were analyzed by one-way ANOVA or *t*’-test. ^*^ *p* < 0.05, ^**^ *p* < 0.01, ^***^ *p* < 0.001.

Previous studies revealed that in macrophages, LXRs are repressors of LPS-induced inflammatory genes, such as IL-1β, IL-6, MCP-1, MMP-13, and iNOS (Ogawa et al., 2005; Ghisletti et al., 2007). mRNA expression levels of inflammatory genes IL-6 and IL-1β were determined by qPCR. These results showed that after stimulation with 200 ng/mL of LPS for 3 h, expression of these genes was significantly increased. However, mRNA levels of these inflammatory genes were dose-dependently decreased by pretreatment with T0901317 for 18 h (Figures 1A,B). Next, we examined protein levels by ELISA and western blot experiments after pretreatment with T0901317 and LPS stimulation for 18 h. Similarly, expression of IL-1β and IL-6 in RAW264.7 macrophages exhibited a decreasing trend in T0901317 groups (Figures 1C,D). As shown in Figures 1E–G, protein expression of COX-2 and iNOS (two typical markers of inflammation) were remarkably upregulated after stimulation with 200 ng/mL of LPS, but dose-dependently repressed following T0901317 treatment. However, the inhibition of T0901317 on IL-1β and IL-6 mRNA levels were attenuated when LXRα/β was silenced (Figures 2A–D). This result suggested that T0901317 reduced LPS-induced inflammatory cytokines levels depended on LXR activation.

### 3.2 T0901317 inhibits TLR4-mediated NF-κB pathway activation

**FIGURE 3:**
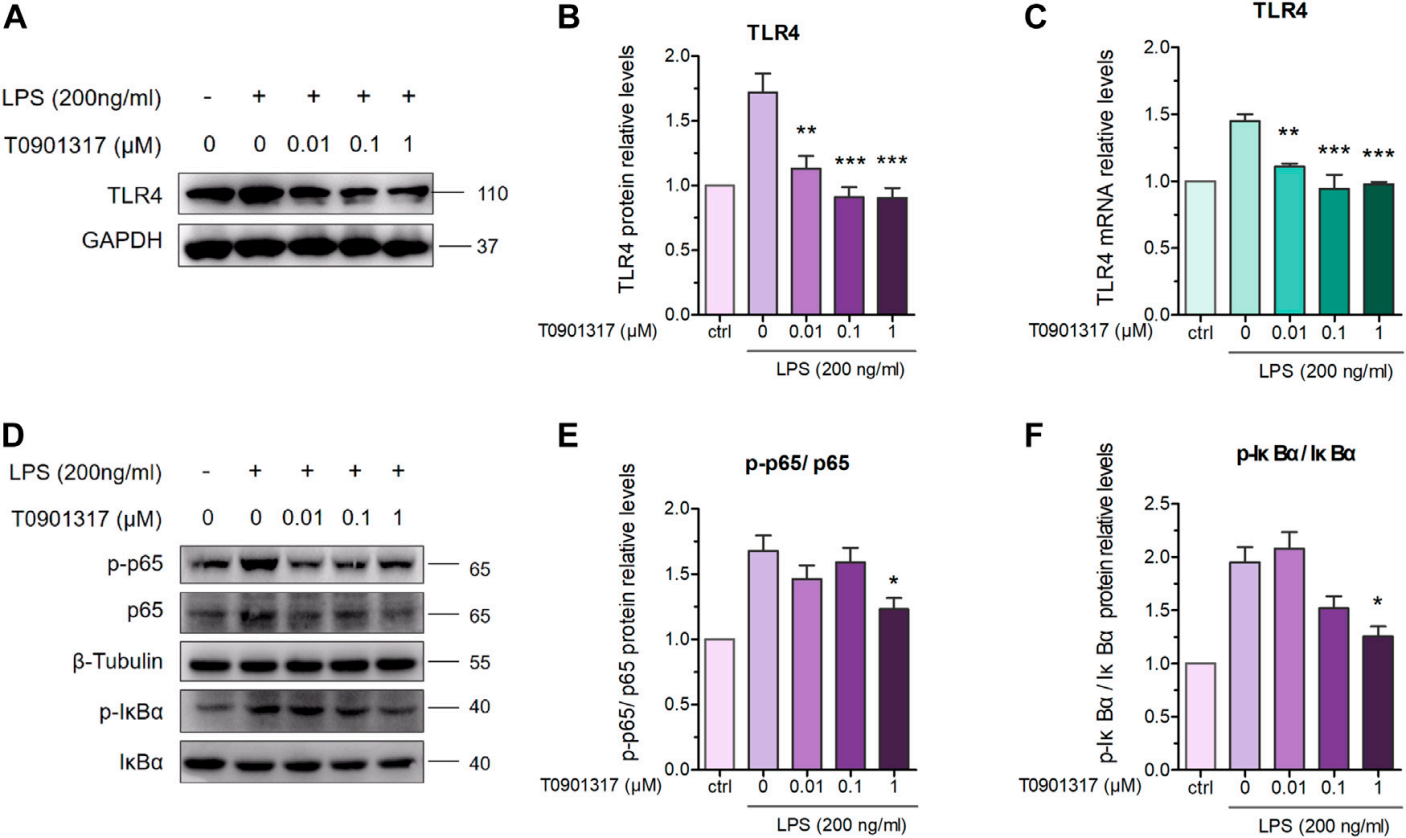
T0901317 inhibits protein levels in TLR4-mediated NF-κB pathway. **(A,B)** RAW264.7 macrophages treated with LPS (200 ng/mL) and T0901317 (0, 0.01, 0.1, or 1 μM) for 18 h, then protein levels of TLR4 were measured by western blot. TLR4 protein level was normalized to GAPDH. **(C)** RAW264.7 macrophages were pretreated with T0901317 (0, 0.01, 0.1, or 1 μM) for 18 h, then stimulated with or without LPS (200 ng/mL) for 3 h. TLR4 mRNA level was measured by qPCR. **(D–F)** RAW264.7 macrophages were pretreated with T0901317 (0, 0.01, 0.1, or 1 μM) for 18 h, then stimulated with or without LPS (200 ng/mL) for 1 h. Phosphorylated IκBα, phosphorylated p65, IκBα and p65 protein levels were measured by western blot. Phosphorylated IκBα protein level was normalized to IκBα, and phosphorylated p65 protein level was normalized to p65, respectively. Similar results were obtained in three independent experiments. Values are expressed as means ± SEM and were analyzed by one-way ANOVA. ^*^ *p* < 0.05, ^**^ *p* < 0.01, ^***^ *p* < 0.001.

TLR4 exerts important regulatory effects on the inflammatory pathway induced by LPS, including NF-κB pathway activation. After stimulation with 200 ng/mL of LPS for 18 h, TLR4 mRNA and protein levels were significantly upregulated; however, T0901317 dose-dependently repressed this upregulation (Figures 3A–C). During TLR4-mediated inflammation, phosphorylation of p65 is necessary (Mitchell et al., 2016). As shown in Figures 3D–F, protein expression of phosphorylated p65 and phosphorylated IκBα were remarkably increased following treatment with 200 ng/mL of LPS for 1 h, indicating that the NF-κB signaling pathway was activated. However, compared with the LPS group, phosphorylation levels of p65 and IκBα proteins were obviously downregulated by T0901317 administration. These results suggest that T0901317 contributed to repression of TLR4-mediated NF-κB pathway activation.

### 3.3 T0901317 affects MyD88 alternative splicing

**FIGURE 4:**
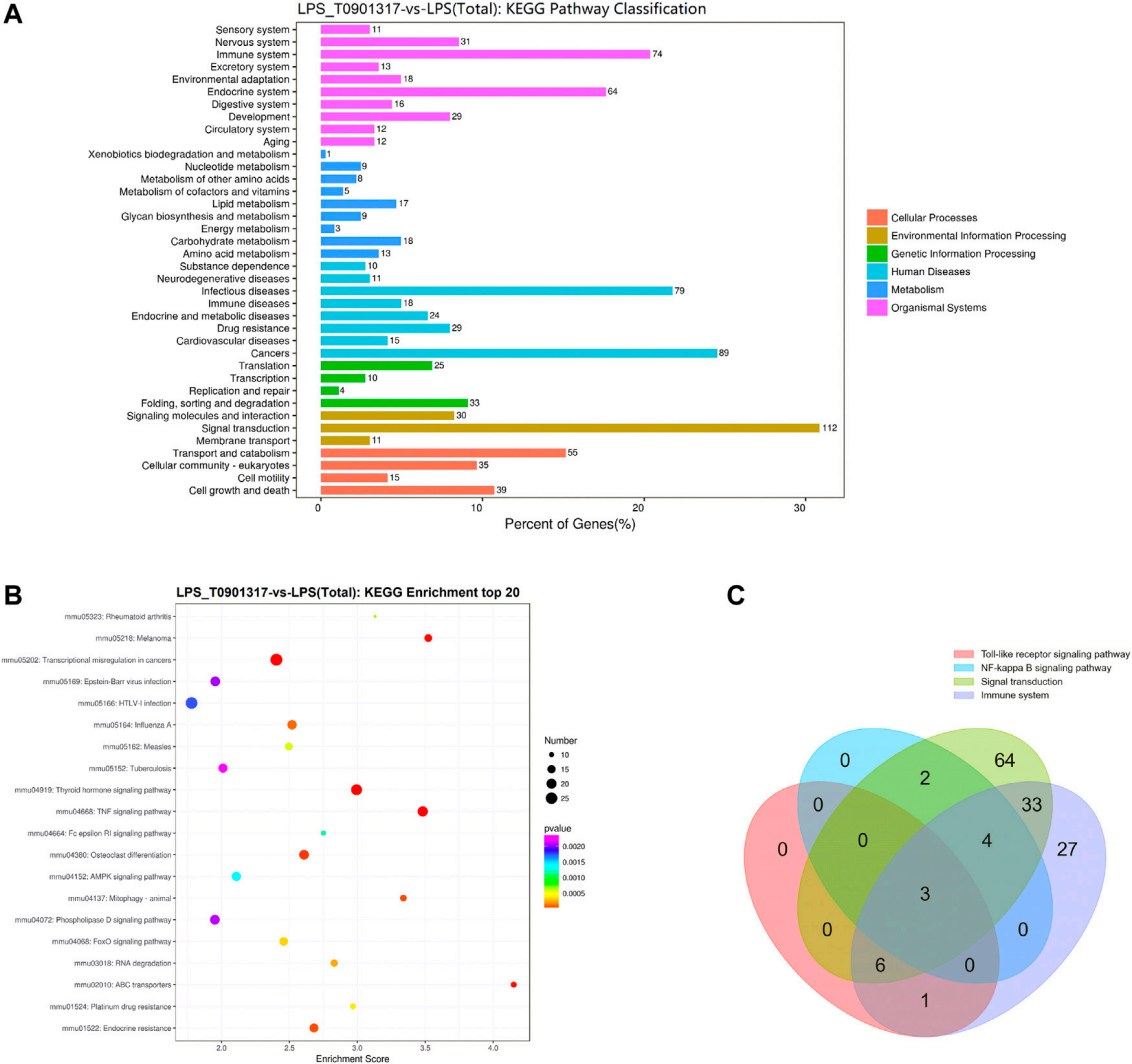
RNA-sequencing analysis of T0901317 treating cells. **(A,B)** Alternative mRNA splicing analysis was performed basing on RNA-seq and KEGG enrichment analysis in LPS/T0901317 vs LPS group were shown here. **(C)** DEGs in four pathways (signal transduction 112 genes, immune system 74 genes, TLR signaling pathway nine genes and NF-κB signaling pathway 10 genes) were subjected to overlapped analysis in a Venn diagram. The number of overlapping genes was shown in the overlapping regions.

Alternative mRNA splicing is involved in inflammation response and occurs in many innate immunity genes, some of which are known to inhibit TLR signaling pathway, such as MyD88 (De Arras and Alper, 2013). RAW 264.7 macrophages treating with LPS or LPS/T0901317 were subjected to RNA sequencing separately to identify the different transcripts of genes regulated by T0901317 (3 samples per experimental group). The Kyoto Encyclopedia of Genes and Genomes (KEGG) pathway analysis of differentially expressed genes (DEGs) between the LPS and LPS/T0901317 revealed that the different genes were significantly enriched in the pathways regulating signal transduction and immune system (Figures 4A,B). Basing on our current results (Figure 3), TLR and NF-κB signaling pathway were considered to be important pathway for T0901317 to inhibit inflammation. To further identify the genes affected by T0901317 for alternative splicing events, the different genes from four pathways (signal transduction 112 genes, immune system 74 genes, TLR signaling pathway 9 genes and NF-κB signaling pathway 10 genes) were subjected to overlapped analysis (Supplementary Table S3). In total, there were three genes (Myd88, Cd40 and Map3k7) overlapped among the four pathways (Figure 4C). This suggested that T0901317 might inhibit inflammation through regulating alternative mRNA splicing of some crucial genes relating to the inflammatory pathway.

**FIGURE 5:**
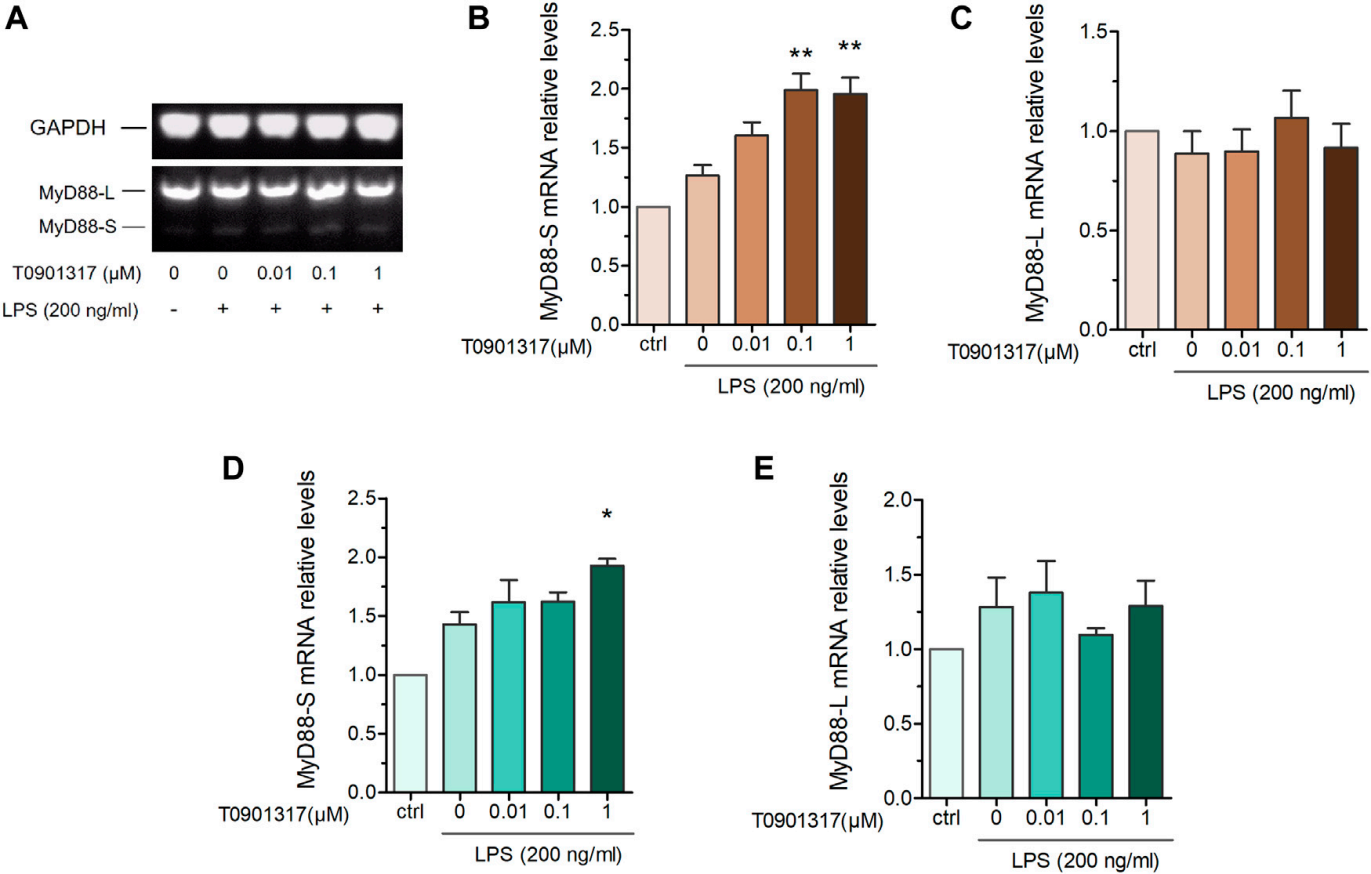
T0901317 affects MyD88 alternative splicing. RAW264.7 macrophages were pretreated with T0901317 (0, 0.01, 0.1, or 1 μM) for 18 h, followed by stimulation with or without LPS (200 ng/mL) for 3 h **(A–C)** mRNA levels of MyD88-S and MyD88-L isoforms were measured by semi-quantitative RT-PCR. **(D,E)** mRNA levels of MyD88-S and MyD88-L isoforms were measured by qPCR. All mRNA levels were normalized to GAPDH. Similar results were obtained in three independent experiments. Values are expressed as means ± SEM and were analyzed by one-way ANOVA. ^*^ *p* < 0.05, ^**^ *p* < 0.01.

MyD88 is an adaptor protein involved in IL-1 receptor- and TLR-induced activation of NF-κB. Interestingly, MyD88-L and MyD88-S exhibit opposing effects on inflammatory pathways, with MyD88-S behaving as a dominant-negative inhibitor of LPS and IL-1 activation (De Arras and Alper, 2013). To further determine the MyD88-splicing mechanism regulated by T0901317, we adopted semi-quantitative PCR and qPCR experiments using specific primers. Our results show that the MyD88-S splicing isoform was dose-dependently increased in T0901317-pretreated groups, while the MyD88-L splicing isoform remained unchanged compared with other groups (Figures 5A–E). This result suggests that the LXR agonist T0901317 inhibited inflammation by regulating alternative splicing of MyD88 and upregulating mRNA expression of the MyD88-S splicing isoform in RAW264.7 macrophages.

### 3.4 Inhibition of SF3A1 attenuates LPS-induced inflammation in coordination with T0901317

**FIGURE 6:**
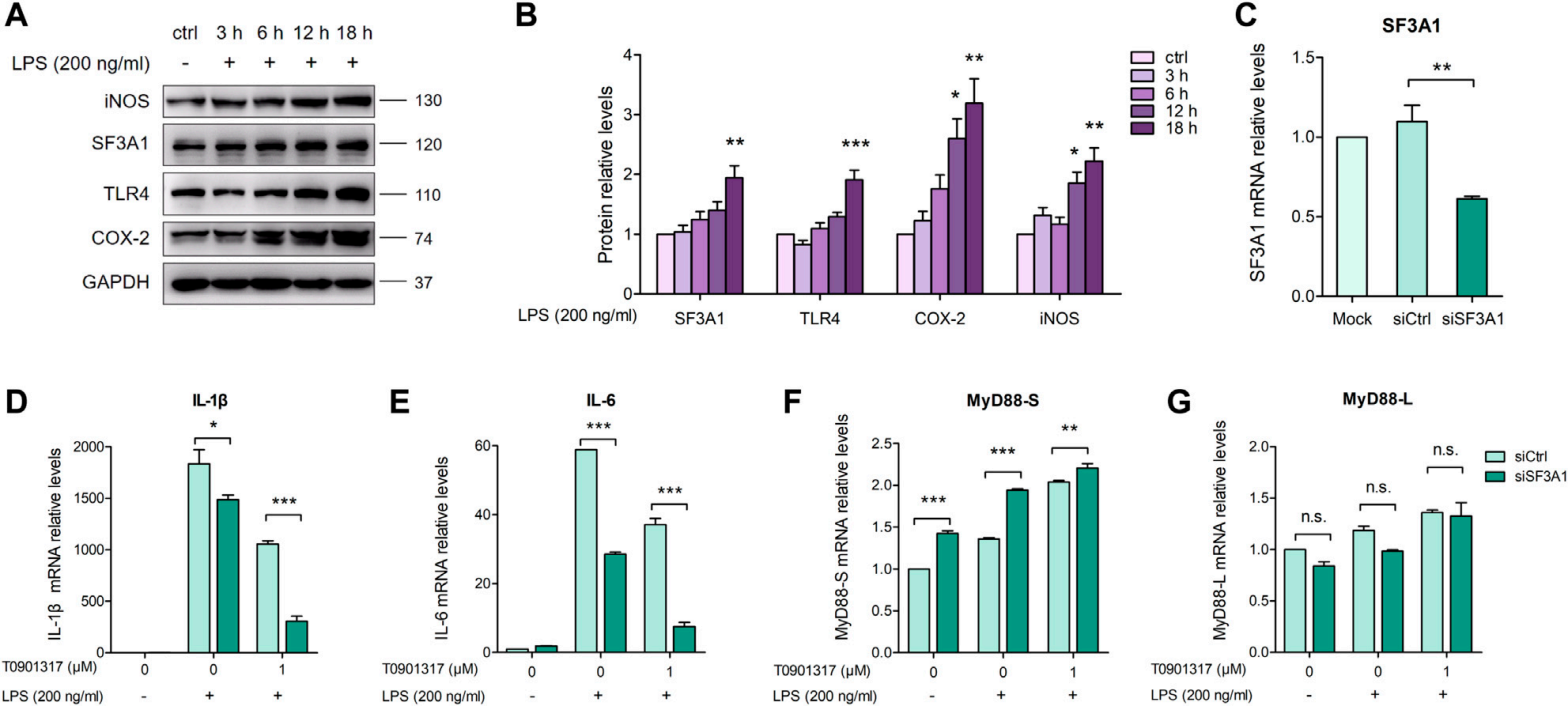
Inhibition of SF3A1 synergistically attenuates LPS-induced inflammation with T0901317. **(A,B)** RAW264.7 macrophages were exposed to LPS (200 ng/mL) for 3, 6, 12, or 18 h, then SF3A1, TLR4, iNOS, and COX-2 protein levels were measured by western blot. **(C–G)** RAW264.7 macrophages were transfected with negative control siRNA or SF3A1 siRNA for 24 h, followed by pretreating with T0901317 (0 or 1 μM) for 18 h, then stimulated with or without LPS (200 ng/mL) for 3 h. SF3A1, IL-1β, IL-6, MyD88-S, and MyD88-L isoform mRNA levels were measured by qPCR. All mRNA and protein levels were normalized to GAPDH. Similar results were obtained in three independent experiments. Values are expressed as means ± SEM and were analyzed by one-way ANOVA. ^*^ *p* < 0.05, ^**^ *p* < 0.01, ^***^ *p* < 0.001.

The mRNA splicing factor SF3A1 can reportedly regulate LPS-induced cytokine production, such as IL-6 and TNFα, by regulating splicing of the TLR4-regulatory gene MyD88 (De Arras and Alper, 2013). We aimed to determine the mechanism by which T0901317 affects MyD88 splicing to inhibit inflammation. First, we stimulated cells with 200 ng/mL of LPS for 3, 6, 12, or 18 h, and evaluated protein levels of TLR-regulatory genes. As shown in Figures 6A,B, protein expression of TLR4, COX-2, and iNOS was notably upregulated by LPS at 18 h, indicating activation of the TLR4-mediated inflammatory pathway. Consistent with this result, stimulation with LPS for different times led to a slight increase of SF3A1 protein. Therefore, our results indicate that SF3A1 is affected by LPS stimulation, followed by alteration of TLR4-regulatory genes and LPS-induced cytokine production.

To further confirm the involvement of SF3A1 in inhibition of inflammation and regulation of MyD88 splicing, siRNA knockdown of SF3A1 (siSF3A1) was performed in macrophages (Figure 6C). As shown in Figures 6D,E, siSF3A1 obviously inhibited LPS-induced mRNA expression of key pro-inflammatory cytokines IL-1β and IL-6. Notably, mRNA expression of pro-inflammatory cytokines was lower in the siSF3A1/T0901317 group compared with the group treated with T0901317 alone. In addition, MyD88-S was increased by siSF3A1 following stimulation with LPS. In the T0901317 and LPS co-treatment group, MyD88-S was slightly upregulated by siSF3A1 (Figure 6F). However, no significant difference in MyD88-L regardless of siSF3A1 or T0901317 treatment (Figure 6G). These results suggest that inhibition of SF3A1 attenuated LPS-induced inflammation by upregulating MyD88-S levels. This conclusion is consistent with findings described in De Arras et al. (2013).

**FIGURE 7:**
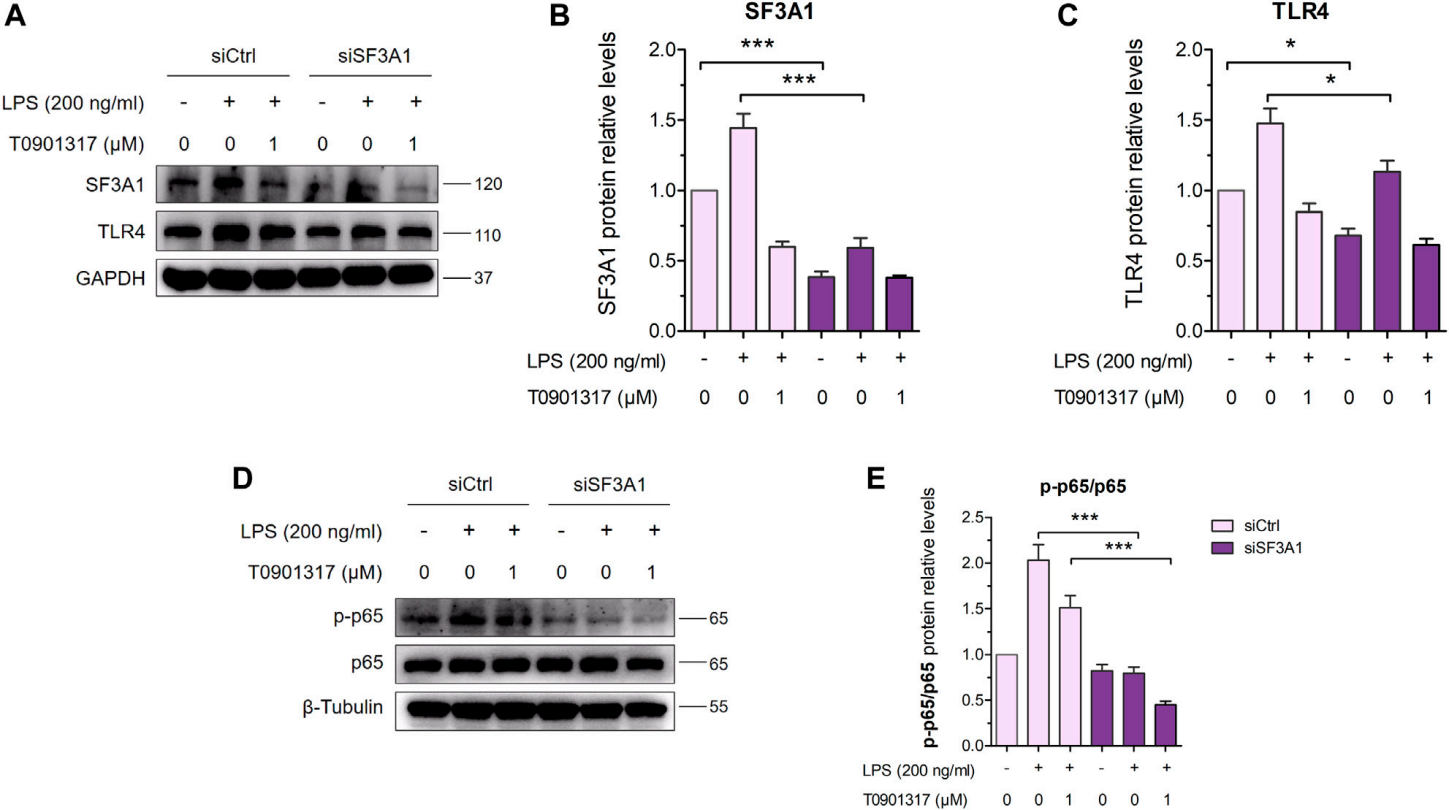
Inhibition of SF3A1 synergistically attenuates inflammation in TLR4-mediated NF-κB pathway. **(A–C)** RAW264.7 macrophages were transfected with negative control siRNA or SF3A1 siRNA for 24 h, followed by treating with or without LPS (200 ng/mL) and T0901317 (0 or 1 μM) for 18 h, then protein levels of SF3A1 and TLR4 were measured by western blot, normalized to GAPDH. **(D,E)** RAW264.7 macrophages were transfected with negative control siRNA or SF3A1 siRNA for 24 h, followed by pretreating with T0901317 (0 or 1 μM) for 18 h, then stimulated with or without LPS (200 ng/mL) for 1 h. Phosphorylated p65 and p65 protein levels were measured by western blot. Phosphorylated p65 protein level was normalized to p65. Similar results were obtained in three independent experiments. Values are expressed as means ± SEM and were analyzed by one-way ANOVA. ^*^ *p* < 0.05, ^***^ *p* < 0.001.

Furthermore, siSF3A1 obviously inhibited LPS-induced protein expression of TLR4 and phosphorylated p65-mediated pathway (Figures 7A–C), indicating that SF3A1 was involved in TLR4-mediated NF-κB pathway activation. However, As shown in Figures 7D,E, the protein level of phosphorylated p65 was even lower in the siSF3A1/T0901317 group compared with the group treated with T0901317 alone. These results suggest that T0901317 treatment and inhibition of SF3A1 had a synergistic anti-inflammatory effect mediated by TLR4 pathway, both could up-regulate MyD88-S.

### 3.5 Over-expression of SF3A1 impairs inflammatory inhibition of T0901317

**FIGURE 8:**
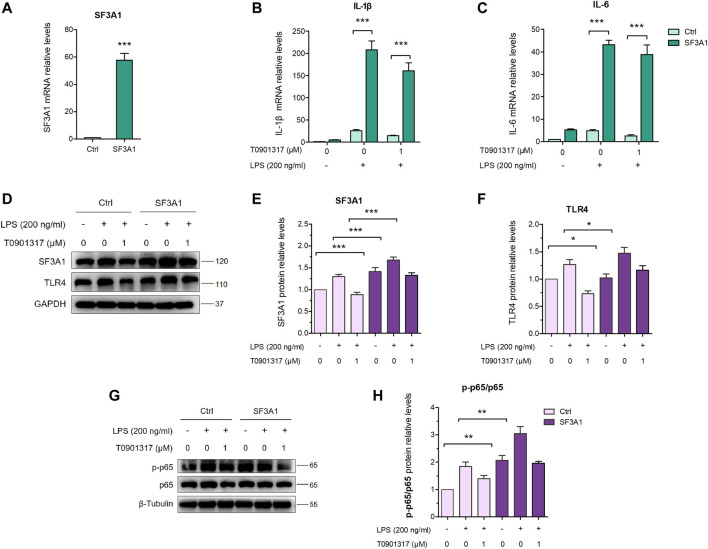
Inhibition of SF3A1 synergistically attenuates inflammation in TLR4-mediated NF-κB pathway. **(A–C)** RAW264.7 were macrophages transfected with or without pCMV3-Flag-SF3A1 plasmid for 24 h, followed by pretreating with T0901317 (0 or 1 μM) for 18 h, then stimulated with or without LPS (200 ng/mL) for 3 h. SF3A1, IL-1β and IL-6 mRNA levels were measured by qPCR. All mRNA levels were normalized to GAPDH. **(D–F)** Cells were transfected with or without pCMV3-Flag-SF3A1 plasmid for 24 h, followed by treating with or without LPS (200 ng/mL) and T0901317 (0 or 1 μM) for 18 h, then protein levels of SF3A1 and TLR4 were measured by western blot, normalized to GAPDH. **(G,H)** RAW264.7 macrophages were transfected with or without pCMV3-Flag-SF3A1 plasmid for 24 h, followed by pretreating with T0901317 (0 or 1 μM) for 18 h, then stimulated with or without LPS (200 ng/mL) for 1 h. Phosphorylated p65 and p65 protein levels were measured by western blot. Phosphorylated p65 protein level was normalized to p65. Similar results were obtained in three independent experiments. Values are expressed as means ± SEM and were analyzed by one-way ANOVA. ^*^ *p* < 0.05, ^**^ *p* < 0.01, ^***^ *p* < 0.001.

As shown in Figures 8A–C, over-expression of SF3A1 significantly increased LPS-induced mRNA expression of key pro-inflammatory cytokines IL-1β and IL-6. However, mRNA levels of pro-inflammatory cytokines were higher in the SF3A1/T0901317 group compared with the group treated with T0901317 alone. In addition, SF3A1 over-expression markedly up-regulated LPS-induced protein expression of TLR4 and phosphorylated p65 (Figures 8D–F), indicating that SF3A1 promoted TLR4-mediated NF-κB pathway activation. However, As shown in Figures 8G,H, the protein level of TLR4 and phosphorylated p65 were slightly increased in the SF3A1/T0901317 group compared with the group treated with T0901317 alone. These results suggest that SF3A1 could accelerate LPS-induced inflammation mediated by TLR4 pathway, and SF3A1 overexpression also partially impaired inflammatory inhibition of T0901317.

### 3.6 T0901317 reduces SF3A1 expression in RAW264.7 macrophages

**FIGURE 9:**
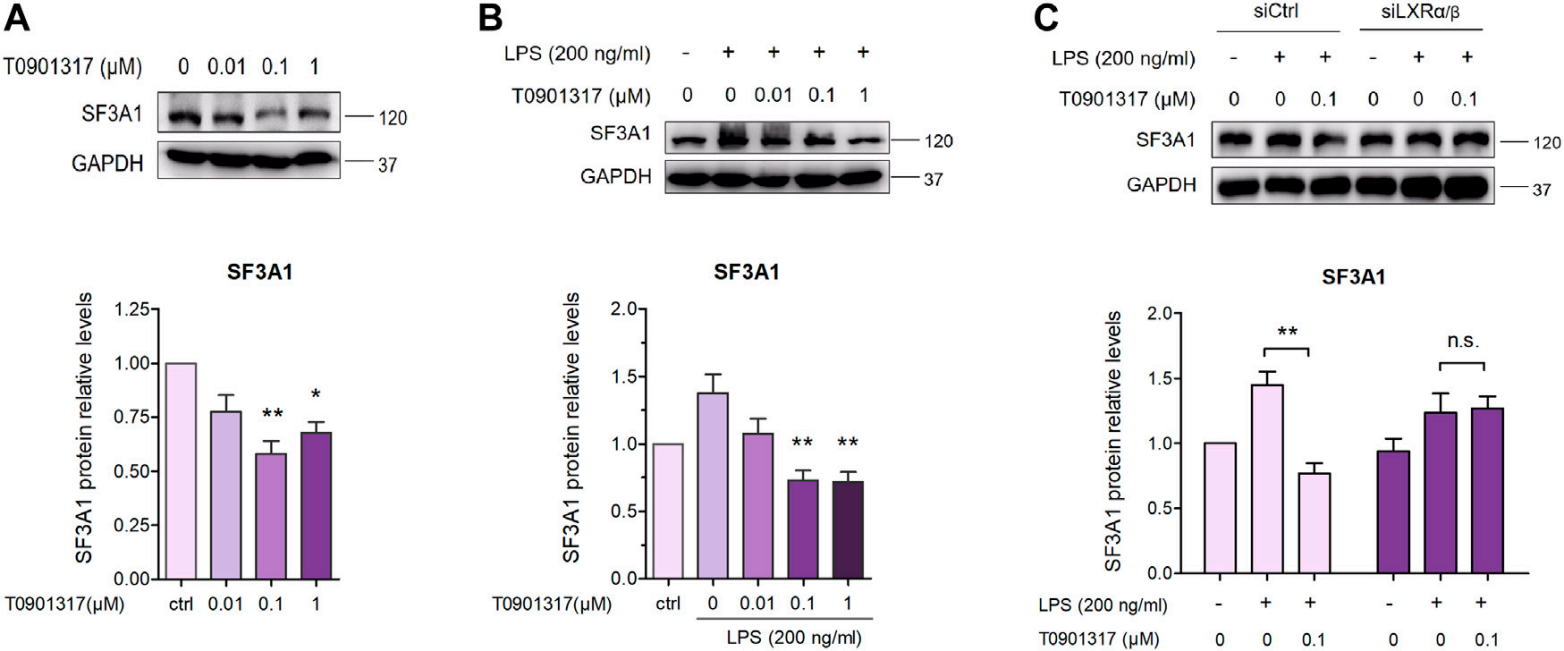
T0901317 decreases SF3A1 protein expression in RAW264.7 macrophages. **(A)** RAW264.7 macrophages were treated with T0901317 (0, 0.01, 0.1, or 1 μM) for 18 h **(B)** RAW264.7 macrophages were exposed to LPS (200 ng/mL) and T0901317 (0, 0.01, 0.1, or 1 μM) for 18 h **(C)** RAW264.7 macrophages were transfected with negative control siRNA or LXRα/β siRNA for 24 h, then exposed to LPS (200 ng/mL) and T0901317 (0 or 0.1 μM) for 18 h SF3A1 protein levels were measured by western blot, normalized to GAPDH expression. Similar results were obtained in three independent experiments. Values are expressed as means ± SEM and were analyzed by one-way ANOVA. ^*^ *p* < 0.05, ^**^ *p* < 0.01.

As previously described (De Arras and Alper, 2013), inhibition of SF3A1 remarkably diminished LPS-induced IL-6 production. Thus, we next examined the impact of LXR agonist T0901317 on SF3A1 protein expression in RAW264.7 macrophages. Treatment with T0901317 resulted in a dose-dependent decrease of SF3A1 protein, regardless of whether stimulating with or without 200 ng/mL of LPS for 18 h (Figures 9A,B). To further confirm that T0901317 decreased SF3A1 protein in an LXR-dependent manner, siRNA knockdown of LXRα/β was carried out. Western blotting analysis showed that the T0901317-induced reduction of SF3A1 was markedly attenuated when LXRα/β was silenced (Figure 9C), indicating that the decrease of SF3A1 expression induced by T0901317 was partially dependent on LXR activation.

## 4 Discussion

**FIGURE 10:**
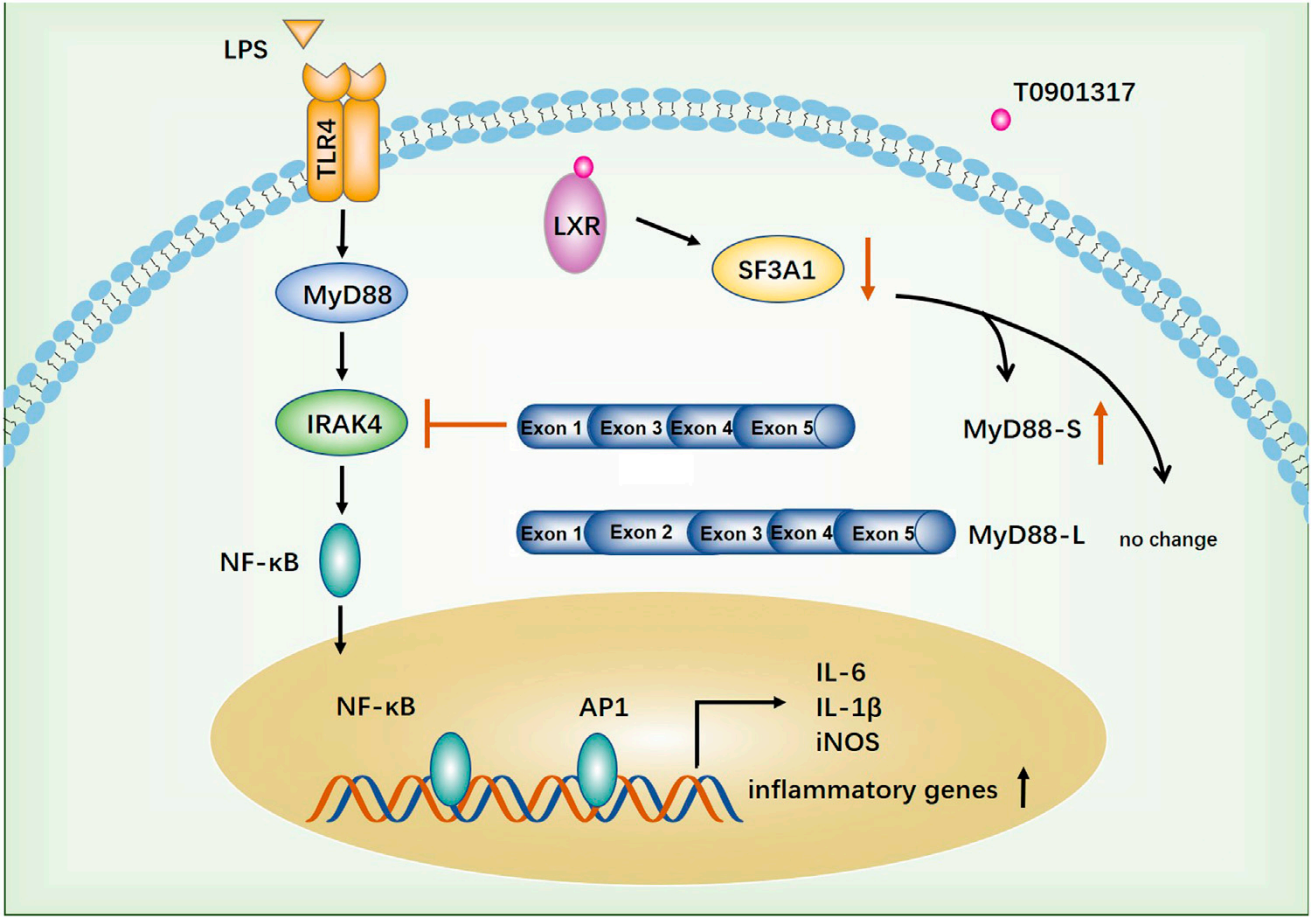
The proposed anti-inflammatory mechanism of T0901317. TLR4-mediated inflammatory pathway was activated by stimulating with LPS. T0901317, a potent LXR agonist, exhibits anti-inflammatory effect through increasing mRNA level of MyD88-S splicing isoform by down-regulating SF3A1 expression, leading to NF-κB mediated inhibition of inflammatory response in RAW264.7 macrophages.

Atherosclerosis, a complex disease characterized by chronic inflammation and injury of the arterial wall, is a leading cause of death worldwide (Benjamin et al., 2018). In the past 2 decades, important roles of macrophages in atherosclerosis and related complications have been elucidated. LXRs generally function as master regulators of lipid metabolism, cholesterol metabolism, development, inflammation, and infection (Jakobsson et al., 2012). Accordingly, recent insights suggest that LXR has great potential as a pharmacological target for anti-atherosclerotic drugs because it could regulate expression of multiple genes (Parikh et al., 2014). In this study, we found that the LXR agonist T0901317 exerted a significant anti-inflammatory effect *in vitro*. Moreover, for the first time, we identified that T0901317 could increase mRNA levels of the MyD88-S splicing isoform by down-regulating SF3A1 expression, leading to NF-κB-mediated inhibition of inflammatory responses in RAW264.7 macrophages. These results indicated that the anti-inflammatory function of LXR may be associated to with alternative splicing of mRNA transcripts of genes involved in TLR4-mediated signaling (Figure 10).

Various compounds that modulate the transcriptional activity of LXRs have shown potent anti-atherosclerotic effects *in vitro* and *in vivo*, such as T0901317, GW3965, GSK 2033, and IMB-170 in our previous study (Jakobsson et al., 2012; Steffensen et al., 2013; Li et al., 2014; El-Gendy et al., 2018). Circulating monocytes of patients with atherosclerosis reportedly elicited stronger pro-inflammatory cytokine and chemokine responses to LPS stimulation compared with those of healthy subjects (Bekkering et al., 2016). Therefore, regression of atherosclerotic lesion formation strongly depends on inhibition of macrophage inflammation (Winkels et al., 2018). In the present study, T0901317 exhibited potent anti-inflammatory activity through TLR4-mediated NF-κB pathway. This finding is consistent with previous studies showing anti-inflammatory activity of LXR in macrophages. For example, LXR activation by GW3965 elicited anti-inflammatory effects by reducing CXCL10 and CCL5 levels in lung macrophages (Higham et al., 2013). In addition to suppressing cytokine expression, LXR also induced emigration of monocyte-derived (CD68^+^) cells from aortic plaques, thus repressing the atherosclerotic development (Feig et al., 2010).

Considerable evidence has emerged indicating that LXRs exert a diversity of regulatory mechanisms on inflammatory inhibition. Transrepression of LXR, which involves SUMOylation, is considered the best-known mechanism by which LXRs inhibit inflammation; however, this view has been challenged by further research (Ghisletti et al., 2007). Benoit et al. (Pourcet et al., 2016) demonstrated LXRs inhibit IL-18 levels through multiple mechanisms, including effects at various IL-18 checkpoints: expression, activation, and bioavailability. In 2015, Ito et al. (2015) demonstrated that the ability of LXRs to inhibit inflammatory gene expression in mice and cells primarily depended on regulation of lipid metabolism *via* transcriptional activation, which can occur in the absence of SUMOylation. They found that LXR activation inhibited TLRs signaling to downstream NF-κB and MAP kinase effectors through ABCA1-dependent changes in membrane lipid organization, followed by disruption of MyD88 and TRAF6 recruitment, which is essential for its activation and downstream signaling transduction (Li et al., 2001). These results suggest that many aspects of the TLR4 pathway, not limited to TLR4 regulation or transrepression of inflammatory genes, may be involved in inhibition of inflammation by LXR activation. The interaction between MyD88 and IRAK triggers IRAK phosphorylation, which is essential for its activation and downstream signaling transduction (Li et al., 2001). TLR4 signaling is a multi-target anti-inflammatory pathway, and it plays a crucial role in occurrence and development in inflammation and atherosclerosis. Considering that MyD88 is a key adaptor in the TLR4 signaling pathway, we further determined the regulatory mechanism by which LXR activation affects MyD88 in the current study. Our results suggest that T0901317 contributed to the repression of LPS-induced NF-κB pathway activation mediated by TLR4. Furthermore, based on our RNA-seq analysis, we found that the inflammatory inhibition attributed to alternative splicing of MyD88 mRNA isoforms, in particular MyD88-S, were dose-dependently increased following T0901317 treatment. This result is consistent with conclusions reported by Janssens et al. (2022) who found that MyD88-S behaved as a dominant-negative inhibitor of LPS-induced inflammation (Janssens et al., 2002).

Alternative mRNA splicing, an important mechanism for the expansion of proteome diversity by creation of multiple protein isoforms, is involved in many pathological processes and diseases (Wong et al., 2018). In 2016, deep RNA-sequencing reported by Lin et al. detected a large number of alternative splicing events in primary human monocyte-derived M1 and M2 macrophages, many of which were relevant to macrophage inflammation (Lin et al., 2016). Therefore, we hypothesized that the LXR agonist T0901317 mediates regulation of alternative splicing during inflammation. It was reported that SF3A1, an RNA-binding protein, could regulate LPS-induced cytokine production by regulating splicing of the TLR4-regulatory gene MyD88 (De Arras and Alper, 2013). Based on our investigation, stimulation with LPS led to a slight increase of SF3A1 protein levels, followed by alteration of TLR4-regulated genes and LPS-induced cytokine production. Subsequently, inhibition of SF3A1 attenuated LPS-induced inflammation, which was attributed to upregulation of MyD88-S levels. Moreover, compared with the group treated with T0901317 alone, mRNA expression of pro-inflammatory cytokines and MyD88-S levels were lower in the siSF3A1/T0901317 group. These results showed that inhibition of SF3A1 and T0901317 had a synergistic anti-inflammatory effect mediated by TLR4. Additionally, over-expression of SF3A1 could accelerate LPS-induced inflammation mediated by TLR4 pathway, and also partially impaired inflammatory inhibition of T0901317. Finally, silencing of LXRα/β markedly attenuated the T0901317-induced reduction of SF3A1, indicating that T0901317 decreased SF3A1 expression by a mechanism partially dependent on LXR activation (Figure 10). These results suggested that SF3A1 could be a potential anti-inflammatory target in LXR regulation. Both nuclear receptors and RNA-binding proteins are important regulatory proteins, but the effect of synergistic regulation between them on inflammation is relatively rare. Therefore, the specific regulatory mechanism of LXR on SF3A1 remains to be further studied.

Lipid accumulation in the arterial intima is the essential cause of inflammatory atherogenesis pathology. The CANTOS trial investigating Canakinumab, a pure anti-inflammatory therapy involving a monoclonal antibody that inhibits IL-1β, revealed a complement-dependent mechanism for lowering low-density lipoprotein cholesterol (Ridker et al., 2017). LXRs contributing to lipid and cholesterol metabolism have attracted recent attention because they also display anti-inflammatory activities. Our results identify a novel anti-inflammatory mechanism for the LXR agonist T0901317 through regulation of MyD88 mRNA alternative splicing associated with the TLR4 signaling pathway. These findings provide an important complement to the anti-inflammatory mechanism of LXR agonists, as well as a new perspective for future development of anti-atherosclerotic drugs.

## Data Availability

The datasets presented in this study can be found in online repositories. The names of the repository/repositories and accession number(s) can be found below: https://www.ncbi.nlm.nih.gov/, PRJNA767351.
