# Supplementary material for: LXR agonist inhibits inflammation through regulating MyD88 mRNA alternative splicing
Source: Front Pharmacol. 2022 Oct 14;13:973612. doi: 10.3389/fphar.2022.973612 (PMC9614042; doi:10.3389/fphar.2022.973612)
Supplement: Supplementary file 3 [file Table1.DOCX]

**Table 1. Primers for real-time quantitative PCR and semi-quantitative RT-PCR**

| **Gene** | **Forward primers** | **Reverse primers** |
| --- | --- | --- |
| mouse GAPDH | 5’- CAATGTGTCCGTCGTGGATCT -3’ | 5’- GTCCTCAGTGTAGCCCAAGATG -3’ |
| mouse IL-1β | 5’- AGAAGCTGTGGCAGCTACCTG -3’ | 5’- GGAAAAGAAGGTGCTCATGTCC -3’ |
| mouse IL-6 | 5’- CTGCAAGAGACTTCCATCCAGTT -3’ | 5’- GAAGTAGGGAAGGCCGTGG -3’ |
| mouse MYD88-L (qPCR) | 5’- CCACCCTTGATGACCCCCTAGGACAAAC -3’ | 5’- GTCTGTTCTAGTTGCCGGATCATCTCCTGCAC -3’ |
| mouse MYD88-S (qPCR) | 5’- GGAGCTGAAGTCGCGCATCGGACAAAC -3’ | 5’- GTCTGTTCTAGTTGCCGGATCATCTCCTGCAC -3’ |
| mouse MYD88 (RT-PCR) | 5’- TTGTTGGATGCCTGGCAGGGGCGCTCTGGC-3’ | 5’- CACGGTCGGACACACACAACTTAAGCCGATAGTC-3’ |
| mouse SF3A1 | 5’-GGACCAGGTTTGTTACCGAGT-3’ | 5’-CCAGTTGGTAACAATGCCATGT-3’ |
| mouse LXRα | 5’-ACAGAGCTTCGTCCACAAAAG-3’ | 5’-GCGTGCTCCCTTGATGACA-3’ |
| mouse LXRβ | 5’-ATGTCTTCCCCCACAAGTTCT-3’ | 5’-GACCACGATGTAGGCAGAGC-3’ |
