# Supplementary figures and images for: LXR agonist inhibits inflammation through regulating MyD88 mRNA alternative splicing
Source: Front Pharmacol. 2022 Oct 14;13:973612. doi: 10.3389/fphar.2022.973612 (PMC9614042; doi:10.3389/fphar.2022.973612)

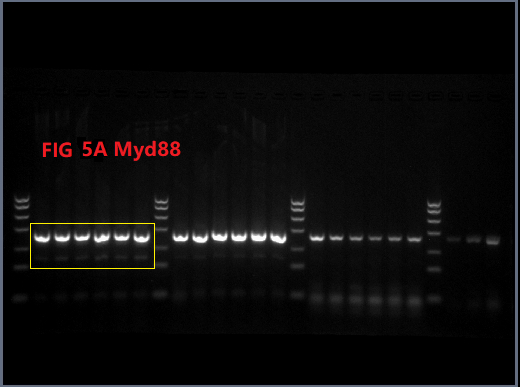

Supplement: Supplementary file 4 [file Image2.TIF]

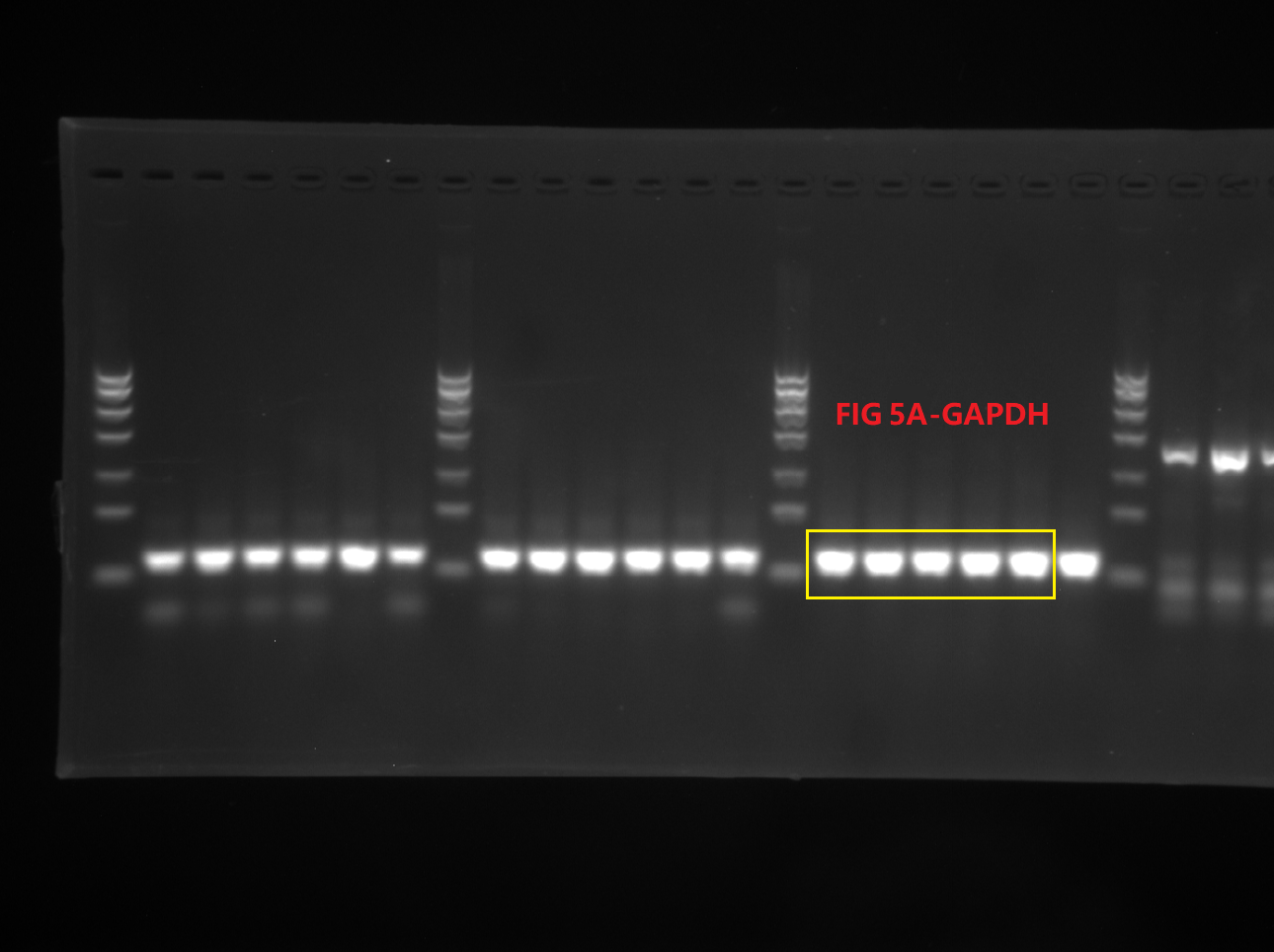

Supplement: Supplementary file 5 [file Image1.TIF]
