# Supplementary material for: LXR agonist inhibits inflammation through regulating MyD88 mRNA alternative splicing
Source: Front Pharmacol. 2022 Oct 14;13:973612. doi: 10.3389/fphar.2022.973612 (PMC9614042; doi:10.3389/fphar.2022.973612)

FIG 1E COX-2

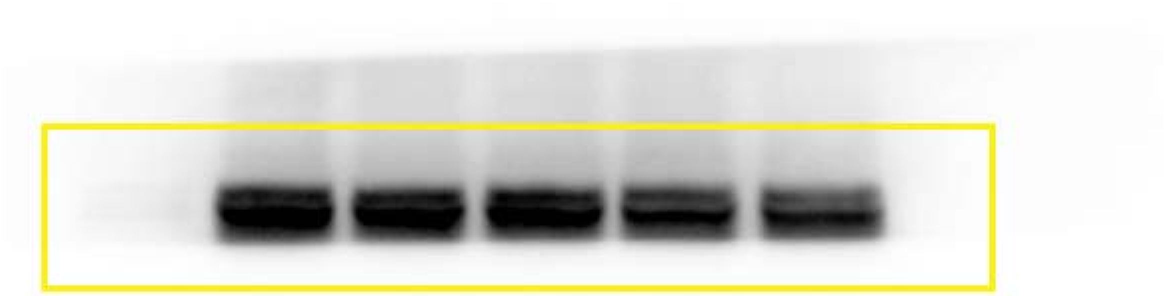

FIG 1E GAPDH

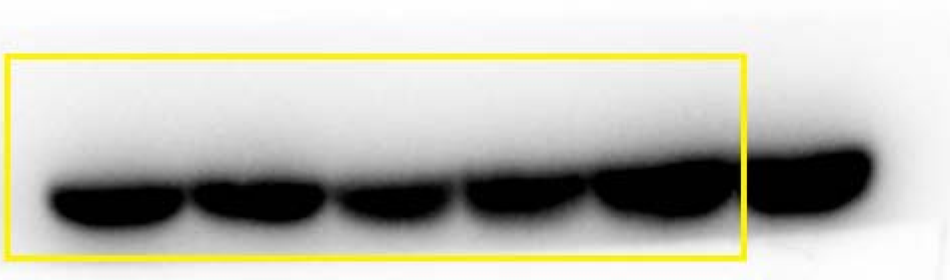

FIG 1E iNOS

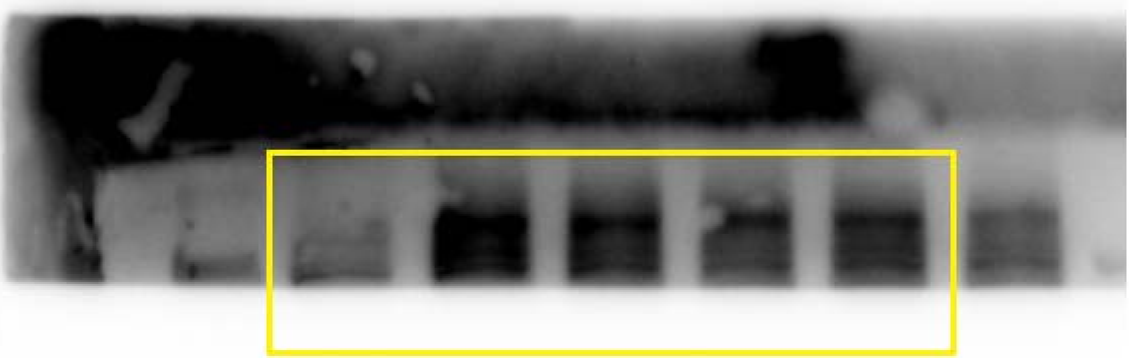

FIG 3A GAPDH

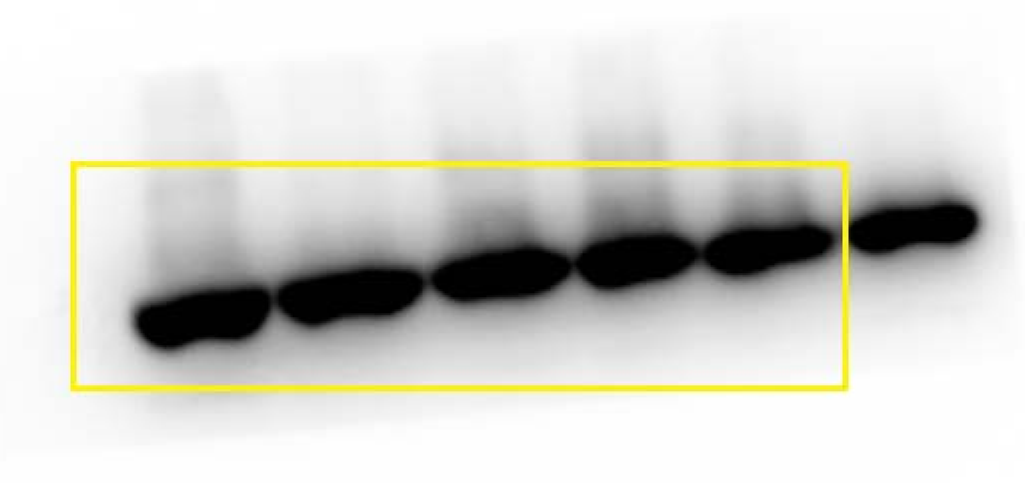

FIG 3A TLR4

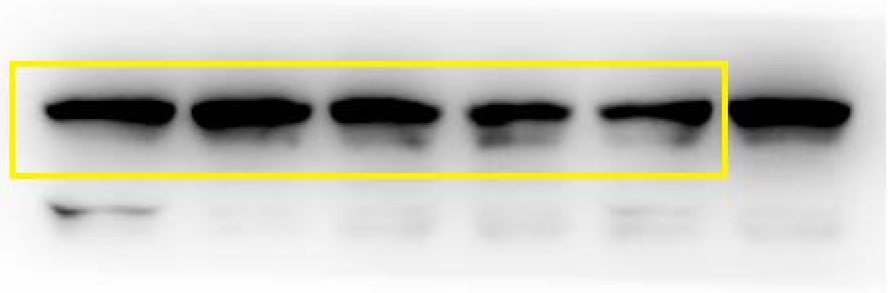

FIG 3C IKBα

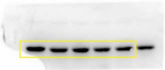

FIG 3C P65

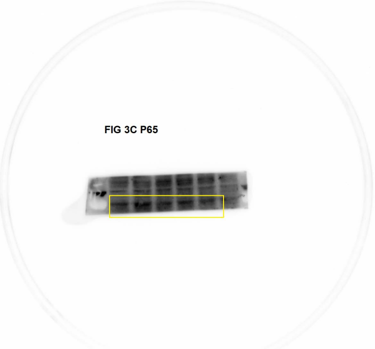

FIG 3C P-IKBα

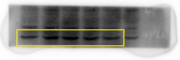

FIG 3C P-P65

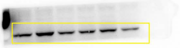

FIG 3C β-Tubulin

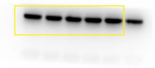

FIG 6A COX-2

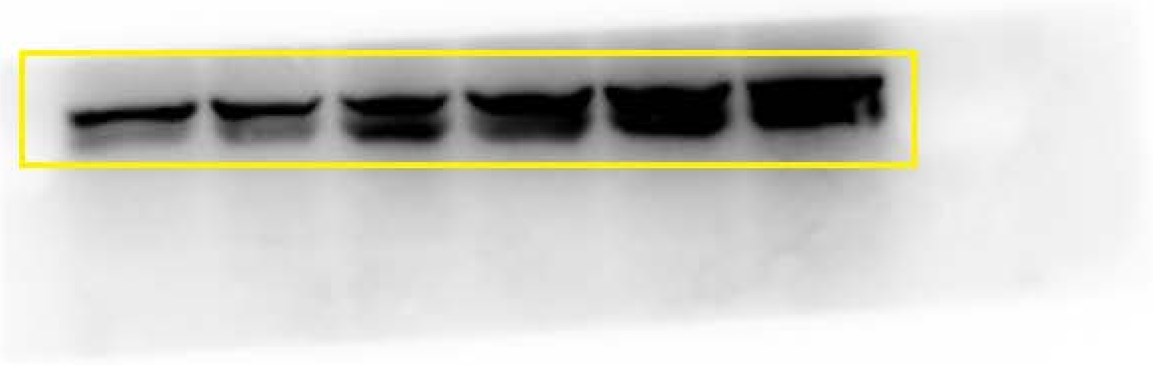

FIG 6A GAPDH

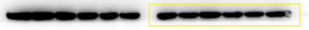

FIG 6A iNOS

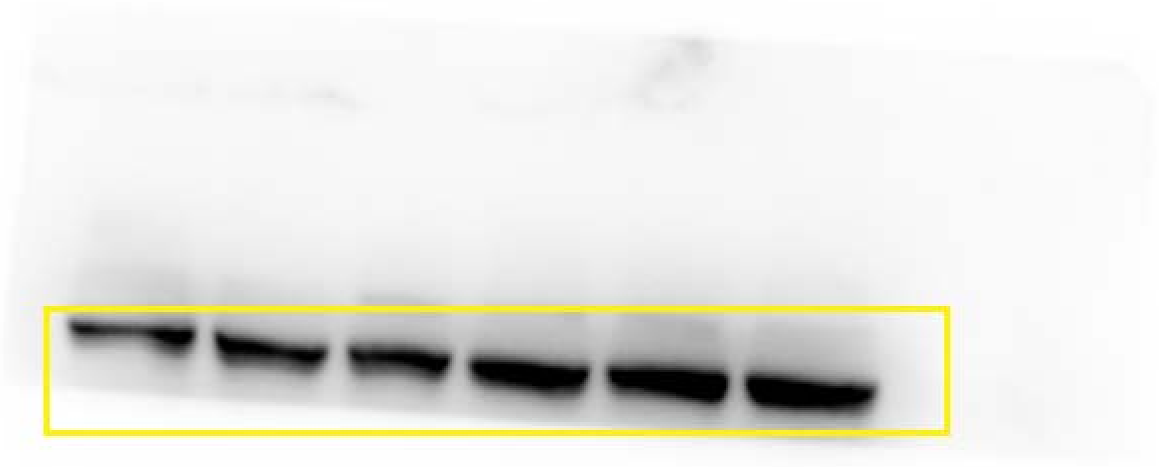

FIG 6A SF3A1

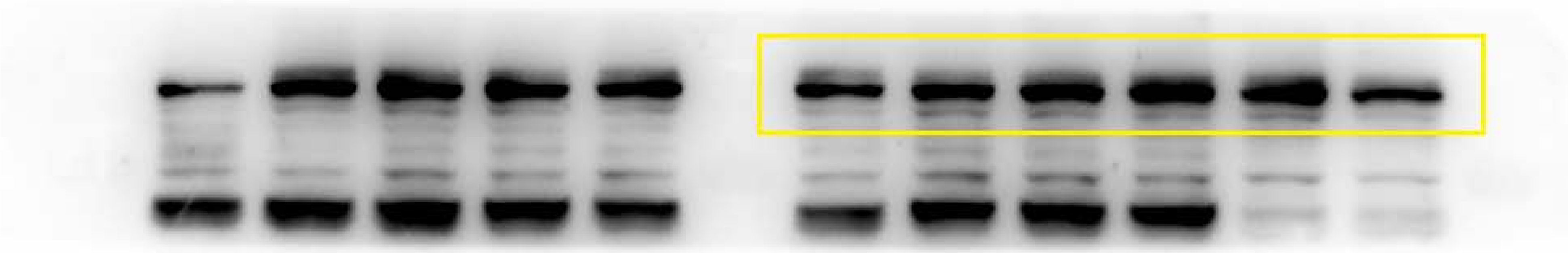

FIG 6ATLR4

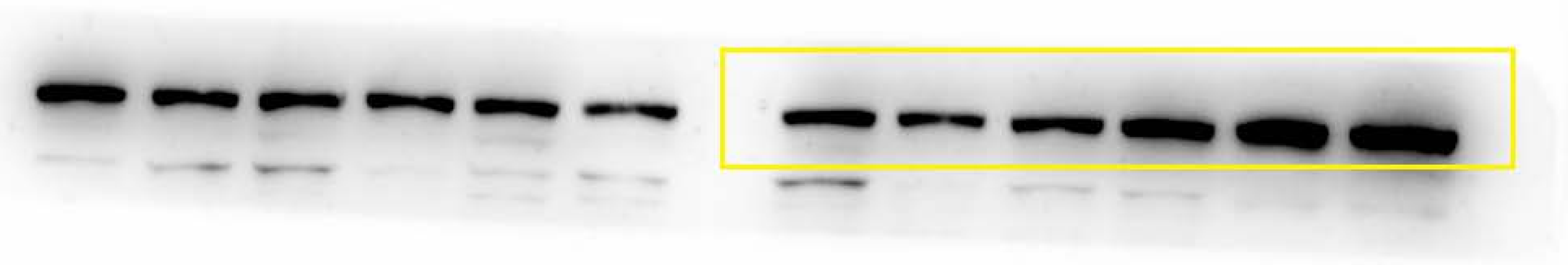

FIG 7A GAPDH

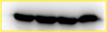

FIG 7A SF3A1

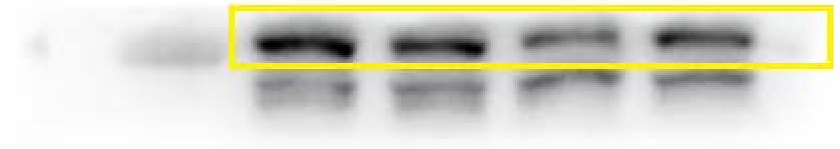

FIG 7B GAPDH

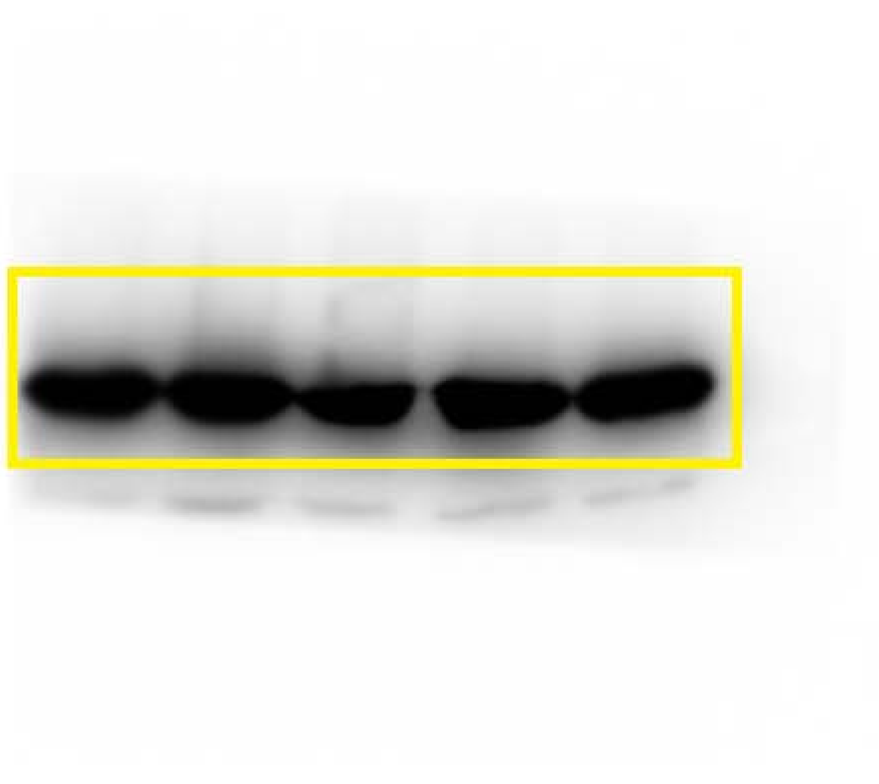

FIG 7B SF3A1

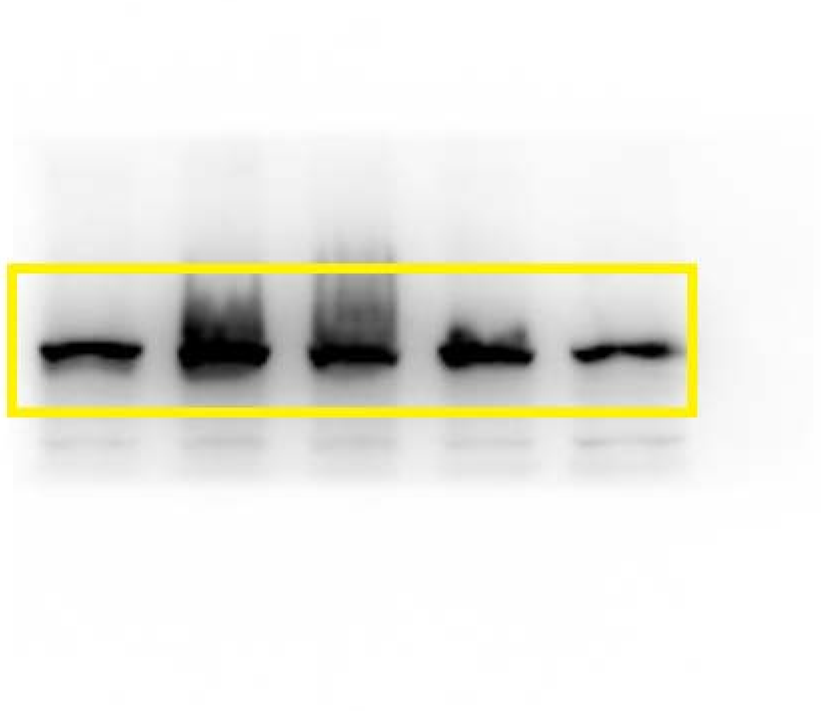

FIG 7C GAPDH

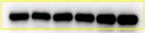

FIG 7C SF3A1

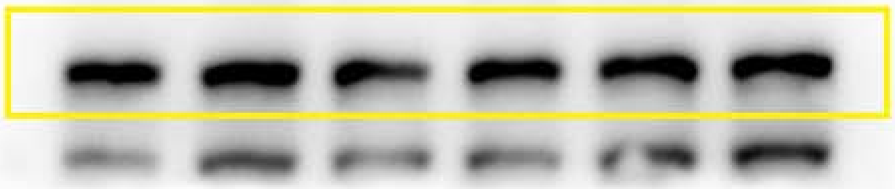

Supplement: Supplementary file 6 [file DataSheet1.PDF]
